# Artesunate induces ferroptosis by inhibiting the nuclear localization of SREBP2 in myeloma cells

**DOI:** 10.7150/ijms.86409

**Published:** 2023-09-18

**Authors:** Laoqi Liang, Yang Liu, Xiaoyan Wu, Yan Chen

**Affiliations:** The Eighth Affiliated Hospital, Sun Yat-sen University, Shen Zhen, Guangdong, China, 518033.

**Keywords:** artesunate, multiple myeloma, ferroptosis, SREBP2, nuclear localization

## Abstract

**Objective:** Multiple myeloma (MM) is an incurable haematological cancer characterized by abnormal proliferation of plasma cells. The promising therapeutic effect of selective inhibitors of nuclear export in MM reveals the broad therapeutic prospects of nuclear localization intervention. Sterol regulatory element binding protein 2 (SREBP2) is a lipid regulatory molecule that has been implicated in the effect of drug therapy for MM. SREBP2 has been reported to be regulated by the antimalarial drug artesunate (ART) through alteration of its nuclear localization and has been shown to inhibit ferroptosis in other tumours. However, the mechanism through which this might occur has not been clarified in MM. Our study aimed to explore whether ART can induce ferroptosis in MM through nuclear localization of SREBP2.

**Methods:** To evaluate whether ferroptosis is induced by treatment with ART in myeloma, we used two types of myeloma cell lines. We first used a series of molecular approaches and other techniques to investigate the impact of ART on cell growth, production of reactive oxygen species (ROS), Fe^2+^ levels, lipid peroxidation and expression of genes related to ferroptosis. Then, we further explored the mechanism through which ferroptosis may occur in these cells and the relationship between ferroptosis and the nuclear localization of SREBP2.

**Results:** Upregulation of ROS, Fe^2+^, and lipid peroxidation as well as inhibition of cell growth were observed in myeloma cells after treatment with ART. Expression of acyl CoA synthase long chain family member 4 (ACSL4) was increased, while glutathione peroxidase 4 (GPX4) expression was reduced in cells treated with ART. ART-induced cell death could be reversed by ferropstatin-1 (Fer-1) and deferoxamine mesylate (DFO). Nuclear localization of SREBP2 in myeloma cells was inhibited, accompanied by downregulation of isopentenyl pyrophosphate (IPP) and GPX4, after treatment with ART.

**Conclusion:** In conclusion, our study demonstrated that the antimalarial drug ART can inhibit nuclear localization of SREBP2, downregulate IPP and GPX4, and eventually trigger ferroptosis in myeloma cells. Through this study, we hope to establish a correlation between nuclear localization pathways and mediation of ferroptosis in myeloma cells and provide an innovative direction for exploration-related therapy.

## Introduction

Multiple myeloma (MM), characterized by abnormal proliferation of plasma cells in the bone marrow^1^, ^2^, is the second most common haematological malignancy (15%)^1^. At present, MM is considered incurable with an unsatisfactory prognosis^3^-^6^. However, the successful development and application of selinexor, an inhibitor of the nuclear export protein XPO1, has shown some therapeutic promise in patients with MM^7^. Selinexor is a new type of medicine that differs from previous treatments used for MM and can achieve greater efficacy in patients with MM^7^. Selinexor inhibits the nuclear export process of a series of tumour suppressor proteins (TSPs) and some growth regulatory proteins^7^, ^8^, reflecting the potential of regulating protein nuclear localization for the treatment of MM.

Recently, modulation of protein nuclear localization has been reported to regulate ferroptosis, an iron-dependent form of cell death that is characterized by unrestricted lipid peroxidation and is associated with the therapeutic effect of treatments for multiple malignant tumours^9^-^11^. For example, the nuclear localization process of tumour suppressor BRCA1 associated protein 1 (BAP1) is promoted by the nuclear import vector IPO2^11^, which results in inhibition of SLC7A11 expression in a deubiquitination-dependent manner and finally promotes the occurrence of ferroptosis^10^, ^12^. E cadherin regulation of the of Yes associated protein (YAP) nuclear localization, through the Hippo signalling pathway, then regulates the synthesis of acyl CoA synthase long chain family member 4 (ACSL4)^12^, a key regulator of ferroptosis. Therefore, inhibition of myeloma cell proliferation may be induced by ferroptosis that occurs via the regulation of nuclear localization of a series of proteins. However, the related effects of this nuclear localization and ferroptosis in MM remain unclear and need to be further explored.

Recent studies have shown that high expression levels of sterol regulatory element binding protein 2 (SREBP2), a key regulator in lipid metabolism, are related to increased drug resistance in MM^13^. Moreover, triggering ferroptosis has been proven to alleviate drug resistance in a variety of malignant tumour cells^14^, ^15^. This suggests that SREBP2 may regulate the drug resistance of MM by regulating ferroptosis. Intriguingly, although the inhibition of ferroptosis has been reported to have a close relationship with SREBP2 upregulation in melanoma^16^, few studies have focused on the interaction between SREBP2 and ferroptosis in myeloma. Increased SREBP2 expression levels promotes an increase in its nuclear localization, which is a prerequisite for it to play regulatory roles^17^-^20^. This finding indicated that there may be a close relationship between the nuclear localization of SREBP2 and the regulation of ferroptosis in myeloma cells.

Some studies have shown that some drugs used initially as anti-parasitic drugs have the ability to regulate the nuclear localization of proteins^21^-^24^. For example, ivermectin, which was used initially to treat strongyloidiasis and lymphatic filariasis^21^, ^22^, was found to have the ability to inhibit the nuclear import process mediated by the importin IPO1^23^. Artesunate (ART), a first-line drug against malaria, has been reported to regulate the nuclear localization of SREBP2 in glioma^24^. Compared with ivermectin, ART is more closely related to haematological malignancies and has been reported to mediate the occurrence of apoptosis in myeloma cells^25^. However, whether ART can mediate other forms of regulatory cell death, such as ferroptosis, has not yet been explored.

Therefore, in the present study, we aimed to explore whether artesunate can induce ferroptosis by regulating the nuclear localization of SREBP2, thus inhibiting the proliferation of myeloma cells.

## Methods

### Reagents and antibodies

The human MM cell lines MM1S (Cat. No. iCell-h291), RPMI8226 (Cat. No. iCell-h183) and human osteosarcoma cell line U2OS (Cat. No. iCell-h218) were obtained from iCell Bioscience (China). The human bronchial epithelial cell line BEAS-2B was purchased from BeNa Culture Collection (Cat. No. BNCC359274). RPMI1640 medium was purchased from Gibco (Cat. No.8121369). Dimethyl sulfoxide (DMSO) (Cat. No.196055) was purchased from MP Biomedicals (USA). Foetal bovine serum (FBS) was obtained from ExCell Bio (Cat. No. FSP500). Penicillin and streptomycin (Pen Strep) (Cat. No. 1541-122) was obtained from Gibco. ART, with a purity of 98%, was purchased from Dalian Meilun Biotechnology Co., Ltd. (No. MB7317) and was dissolved in DMSO for this study. Ferrostatin-1 (Fer-1) (Cat. No. HY-100579), Z-VAD-FMK (Cat. No. HY-16658B), necrostatin-1 (Nec-1) (Cat. No. HY-15760), 3-methyladenine (3-MA) (Cat. No. HY-19312), deferoxamine mesylate (DFO) (Cat. HY-B0988) and GSK343 (Cat. No. HY-13500) were obtained from MedChemExpress (MCE). Antibodies against SREBP2 (ab30682, polyclonal, 1:1000 and 1:200 for Western blot and immunofluorescence techniques, respectively), HRP anti-rabbit IgG antibody (Cat. No. ab288151, 1:2000) and goat anti‑rabbit IgG H&L secondary antibodies (Alexa Fluor® 488, Cat. No. ab150077, 1:200) were purchased from Abcam. Antibodies against GPX4 (Cat. No. #52455, 1:1000) and GAPDH (Cat. No. #2118, 1:1000) were obtained from Cell Signaling Technology. Antibodies against ACSL4 (Cat. No. sc-365230, 1:1000) was purchased from Santa Cruz Biotechnology.

### Cell culture, treatment and transfections

MM1S, RPMI8226, BEAS-2B and U2OS cells were maintained in RPMI 1640 medium supplemented with 10% FBS, 100 U/ml penicillin and 100 mg/ml streptomycin and were incubated at 37°C with 5% CO_2_. Exponentially growing cells were treated with DMSO (0.1%), ART, Fer-1, Z-VAD-FMK, Nec-1, 3-MA and DFO at various concentrations and in different combinations.

The small interfering RNA (siRNA) against SREBP2 were designed and synthesized by RiboBio (RiboBio, Guangzhou, China), and their sequences were as follows: Sequence 1, GATGCCTACTCTTCTCTTA; sequence 2, CAAGGAGAGTCTATACTGT; sequence 3, CGAGCACACTGGTTGAGAT. For transfection, siRNAs (50 nM final concentration), were transfected into appropriate cells using Lipofectamine 3000 (Thermo Fisher Scientific, USA),48 hours after transfection cell pellets were collected and subjected to RNA isolation and immunoblot analysis.

### Cell viability assays

MM1S and RPMI8226 cells were seeded in 96-well plates at a density of approximately 6 × 10^4^ cells per well. BEAS-2B and U2OS cells were seeded in 96-well plates at a density of approximately 1 × 10^4^ cells per well. After treatment for 48 hours, as indicated, 10 µl Enhanced Cell Counting Kit-8 (Cat. No. C0043, Beyotime, Beijing, China) was added to each well and the plates were incubated at 37°C for 2 hours. After incubation, the absorbance value (OD) of each well was measured at 450 nm using a microplate reader (Thermo Fisher Scientific, USA), and the relative OD ratio was calculated and used to represent the viability of the cells.

### Iron measurement

Cells were seeded at a density of 6× 10^4^ cells per well in glass bottom cell culture dishes and incubated with different treatments for 48 hours. After incubation, the culture medium was discarded, and the cells were washed with RPMI 1640 3 times. Then, RPMI 1640 containing 1 µM FerroOrange (Dojindo, Japan, Cat. F374) was added and the cells were incubated for 30 minutes at 37°C in the dark. Finally, the Fe^2+^ content in the cells was analysed using a fluorescence microplate reader (ex 543 nm, em 580 nm) (Tecan Trading AG, Switzerland). In addition, focal images were acquired with a Zeiss inverted LSM 780 laser scanning confocal microscope (Zeiss) using a 100x/1.4 DIC Plan-Apochromat oil immersion objective. Three representative fields were captured for each condition using identical exposure times. Images were obtained with a Cy3 filter (excitation and emission wavelengths 514 and 525-596 nm, respectively). The resolution of the obtained images is 1024×1024 pixels, with a pixel depth of 8 bits, a pinhole size of 1 Airy unit, and a line averaging of 2.

### Estimation of lipid peroxidation

To measure lipid peroxidation in cells, we used a peroxidation malondialdehyde (MDA) assay kit (Cat. No. S0131M, Beyotime Biotech, China) according to the manufacturer's protocol. In brief, after 48 hours of incubation with various treatments, cells were harvested by centrifugation at 1,000 rpm at 4°C for 5 minutes. The supernatant was collected, and thiobarbituric acid (TBA) reagent was added at a ratio of 1:2, after which the mixture was heated at 100°C for 15 minutes. After heating, the mixture was cooled to room temperature and centrifuged at 1000 g at room temperature for 10 minutes. After centrifugation, the supernatant was removed, and the absorbance was measured at 532 nm using an enzyme-labelled instrument. At the same time, intracellular lipid hydroperoxide levels were measured using a selective Liperfluo probe whose concentration was set at 10 μM (Ex = 532 nm, Em = 535-650 nm) and a Zeiss inverted LSM 780 laser scanning confocal microscope (Zeiss) using a 100x/1.4 DIC Plan-Apochromat oil immersion objective. Three representative fields were captured using identical exposure times for each experimental condition. Images were obtained with the FITC filter (ex 488 nm, em 525 nm). The images were obtained at a resolution of are 1024×1024 pixels with a pixel depth of 8 bits, a pinhole size of 1 Airy unit, and a line averaging of 2.

### Measurement of reactive oxygen species (ROS)

After treatment for 48 h, cells were harvested and plated in 6-well plates at a density of 6 × 10^4^ cells/well. Then, all cells were suspended in 1:1000 DCFH-DA (Cat. No. S0033M, Beyotime Biotech, China) and incubated at 37°C for 20 min. After incubation, the cells were washed with RPMI 1640 medium 3 times. Finally, ROS were detected using a Spark multimode microplate reader (Tecan Trading AG, Switzerland). In addition, cells were analysed by confocal microscopy using a Zeiss inverted LSM 780 laser scanning confocal microscope (Zeiss) and a 40x/1.4 DIC Plan-Apochromat oil immersion objective. Three representative fields were captured using identical exposure times for each experimental condition. Images were obtained at a resolution of 1024×1024 pixels with a pixel depth of 8 bits, a pinhole size of 1 Airy unit, and a line averaging of 2 using an FITC filter (ex 488 nm, em 525).

### Quantitative real-time PCR

Total RNA was extracted using TRIzol reagent (Cat. No. T9424, Sigma, USA) according to the manufacturer's protocol. RNA was spectrophotometrically quantified, and equal amounts (1 μg) were reverse transcribed for cDNA synthesis using the Evo M-MLV RT Kit with gDNA Eraser (Cat. No. AG11728, Accurate Biology, China). qPCR analysis was performed on a Roche LightCycler® 480 Real-time fluorescence quantitative PCR instrument with SYBR® Green Premix Pro Taq HS qPCR Kit II (Rox plus) (Cat. No. AG11719, Accurate Biology, China) in a final volume of 10 μl. Each sample was amplified in triplicate. Gene expression levels were calculated using the 2^-ΔΔCt^ method and were normalized to GAPDH expression. The primers used for qRT‒PCR were as follows: human GPX4 forward, 5'-ACAAGAACGGCTGCGTGGTGAA-3'; human GPX4 reverse, 5'-GCCACACACTTGTGGAGCTAGA-3'; human ACSL4 forward, 5'-CATCCCTGGAGCAGATACTCT-3'; human ACSL4 reverse, 5'-TCACTTAGGATTTCCCTGGTCC-3'; human SREBP2 forward, 5'-CTCCATTGACTCTGAGCCAGGA-3'; human SREBP2 reverse, 5'-GAATCCGTGAGCGGTCTACCAT-3'; human GAPDH forward, 5'-GTCTCCTCTGACTTCAACAGCG-3'; human GAPDH reverse, 5'-ACCACCCTGTTGCTGTAGCCAA-3'.

### Western blotting

Cytosol and nuclear proteins were extracted using cytosol/nuclear protein isolation kits (Cat. No. KGP1100, KeyGEN Biotech. Co., Ltd, Nanjing, China) according to the manufacturer's instructions. Cell proteins were extracted using RIPA buffer (Cat. No. P0013B, Beyotime Biotech, China) according to the manufacturer's protocol. The concentration of the extracted protein was measured using BCA protein kits (Cat. No. No. P0012, Beyotime Biotech, China). SDS‒PAGE was conducted to separate the proteins, and separated proteins were transferred to PVDF membranes. After blocking with 5% milk, membranes were fixed overnight at 4°C in a mixture that contained primary antibodies: anti-SREBP2 (1:1000), anti-GAPDH (1:1000), anti-GPX4 (1:1000) or anti-ACSL4 (1:1000). The next day, the membranes were washed and then incubated with an HRP anti-rabbit IgG antibody (1:2000) at room temperature for 1 h. The membranes were visualized using hypersensitive ECL (Cat. No. P0018FS, Beyotime Biotech, China), and the data were collected and analysed using Image J software.

### Immunofluorescence

After undergoing different treatments, cells were fixed with 4% paraformaldehyde (Cat. No. P0099, Beyotime Biotech, China) and washed with phosphate buffer saline (PBS) (Gibco) 2 times. Then, the cells were collected and permeabilized with 0.3% Triton X-100 (Cat. No. ST797, Beyotime Biotech, China) on glass slides (CITOGLAS, China) for 20 min. After washing with PBS 3 times, the cells were incubated in 5% BSA for 30 minutes. Then, the cells were incubated with an antibody against SREBP2 overnight at 4°C. The next day, the cells were incubated with an Alexa Fluor® 488 goat anti-rabbit IgG (H&L) secondary antibody for 1 hour at 37°C. The cells were washed with phosphate-buffered saline supplemented with 0.1% Tween 20 (Cat. No. ST825, Beyotime Biotech, China) (PBST), stained with DAPI and examined under a Zeiss inverted LSM 780 laser scanning confocal microscope (Zeiss).

### Measurement of isopentenyl pyrophosphate (IPP)

The concentration of IPP was measured according to the Human IPP ELISA Kit instructions (Cat. No. KWN216841, KEVINO BIOLOGICAL TECHNOLOGY, China). In brief, the supernatants of treated myeloma cells were collected using RIPA buffer according to the manufacturer's protocol. The levels of IPP in the supernatants were then assessed using enzyme-linked immunosorbent assay (ELISA). HRP-conjugate reagent was added to the experimental wells, and the plates were incubated for 60 minutes in the dark at 37°C. After washing with 1X wash solution, chromogen solutions A and B were added to each well, followed by another incubation for 15 min in the dark at 37°C. Lastly, stop solution was added to each well, and the optical density of each well at 450 nm was read within 15 minutes using a microtiter plate reader. The concentration of IPP in all samples was calculated based on the concentration and absorbance of the standard.

### Statistical analysis

The data was analyzed using Graphpad statistics software. The results were presented as mean ± SE. Comparison between two groups was performed using the t-test. Variations were considered statistically significant when P < 0.05.

## Results

### ART-induced cell death in myeloma cells could be rescued by the ferroptosis-specific inhibitor ferrostatin-1 (Fer-1)

**Figure 1:**
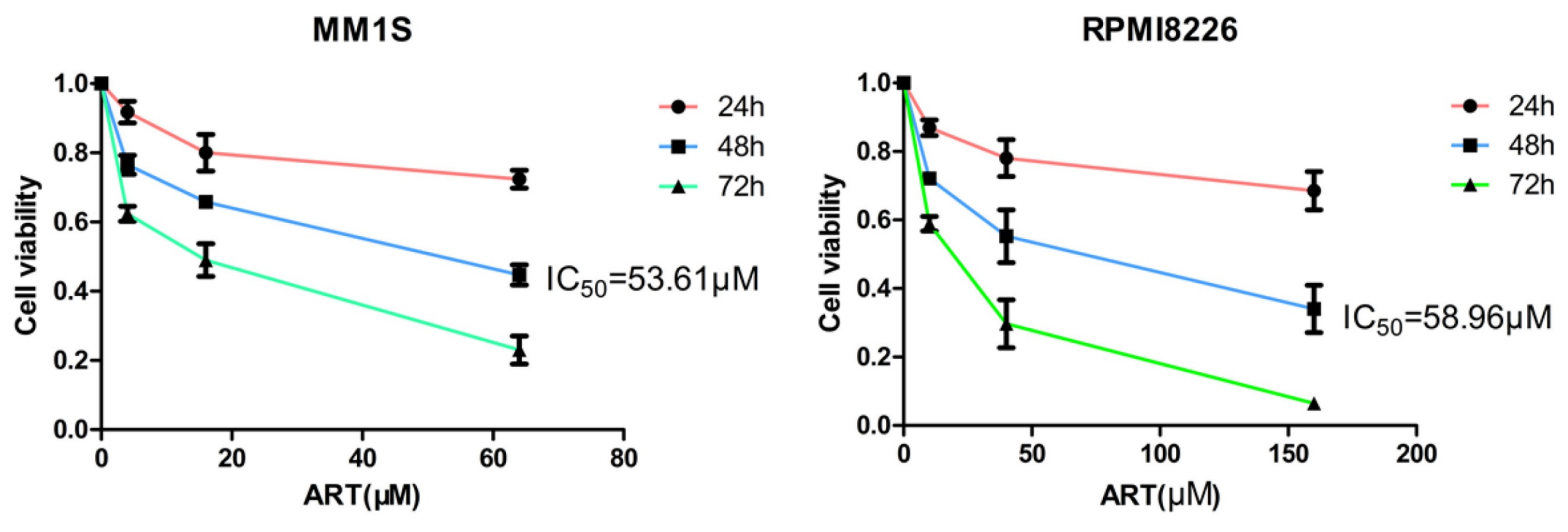
** ART-induced cell death in myeloma cells.** MM1S (left) and RPMI8226 (right) cells were treated with ART for 48 h, and cell viability was assessed using the CCK-8 assay.

**Figure 2:**
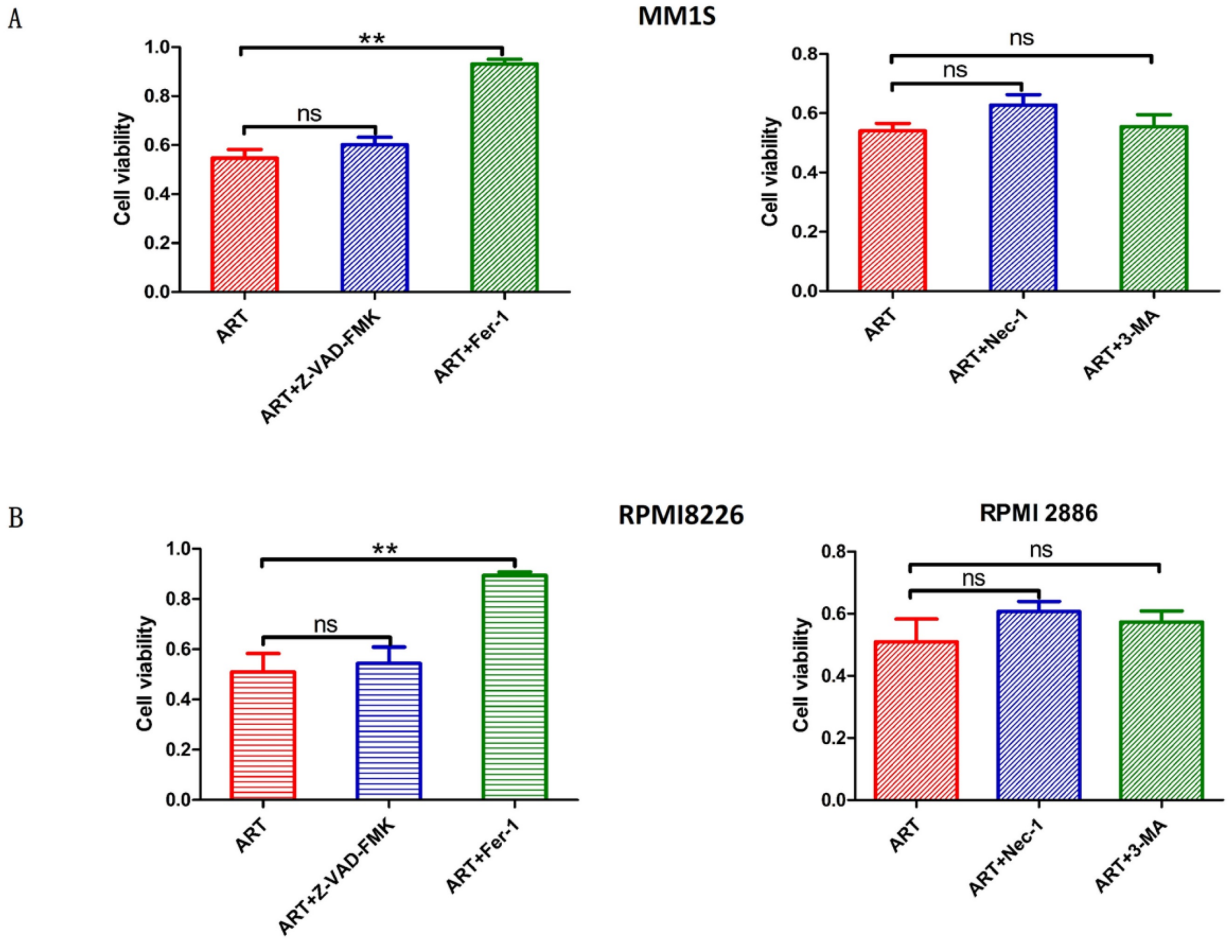
** Evaluation of the impact of treatment with different specific cell death inhibitors on ART-induced cell death in myeloma cells.** MM1S (A) and RPMI8226 (B) were treated with ART (40 µM) in combination with Z-VAD-FMK (10 µM), Fer-1 (2.5 µM), Nec-1 (30 µM) or 3-MA (10 µM) for 48 h, then cell viability was assessed by CCK-8 assay. * P < 0.05, ** P < 0.01, compared with the ART group (40 μM), n=3. Data are presented as the mean ± SE.

Different types of regulatory cell death can be distinguished by treating cells with different specific cell death inhibitors and measuring the subsequent variations in cell viability^26^. Previous studies have reported that ART can trigger apoptosis in myeloma cells^27^, ^28^, but other studies have also noted that ART can trigger nonapoptotic regulatory cell death^29^. Notably, the different concentrations of ART were used in these studies^27^-^29^; we speculate that the type of cell death induced by ART treatment may depend on the concentration of ART used. Therefore, we wanted to explore more types of cell death induced by treatment with ART. First, we evaluated the viability of MM1S and RPMI8226 cells after treatment with different concentrations of ART for 48 hours and found that their IC_50_ values were 53.61 μM and 58.96 μM, respectively (Fig. 1). Then, we treated MM1S and RPMI8226 cells with a 40 μM ART, a concentration that was less than their initial IC_50_, with or without the pancaspase inhibitor Z-VAD-FMK, which can inhibit apoptosis. After 48 hours of incubation, we found that the viability of the cells in the two groups was approximately equal (Fig. 2A, B-Left), indicating that ART may induce a type of nonapoptotic cell death in myeloma cells. To further confirm the specific type of cell death induced by ART, we sequentially treated two myeloma cell lines with ART and a series of specific cell death inhibitors, including ferrostatin-1 (Fer-1, ferroptosis-specific inhibitor), necrostatin-1 (Nec-1, cell necrosis-specific inhibitor) and 3-methyladenine (3-MA, autophagy-specific inhibitor) (Fig. 2A, B-Right). The results showed that the viability of the two myeloma cell lines could be rescued by Fer-1, indicating that ART may induce ferroptosis in myeloma cells.

To further evaluate the effect of ART on cells, we performed a similar analysis in two additional cell lines whose IC_50_ were greater than MM1S and RPMI8226 and thereby considered resistant to ART (Supplementary Fig. S1A, B).After treatment with the same drug concentration as in the present study (ART=40 μM), both the human bronchial epithelial cell line BEAS-2B (IC_50_=63.47μM) and the human osteosarcoma cell line U2OS (IC_50_=70.74μM) show reduction in cell viability (Supplementary Fig. S1A, B). Subsequently, we treated BEAS-2B and U2OS cell lines with Z-VAD-FMK (apoptosis-specific inhibitor) and Fer-1 (ferroptosis-specific inhibitor), respectively, and found that the former, rather than the latter, could rescue ART-mediated proliferation inhibition (Supplementary Fig. S2A, B). These results suggest that ART induces apoptosis rather than ferroptosis in cell lines resistant to ART.

### Alteration of Fe^2+^ levels related to ART-induced cell death in myeloma cells

**Figure 3:**
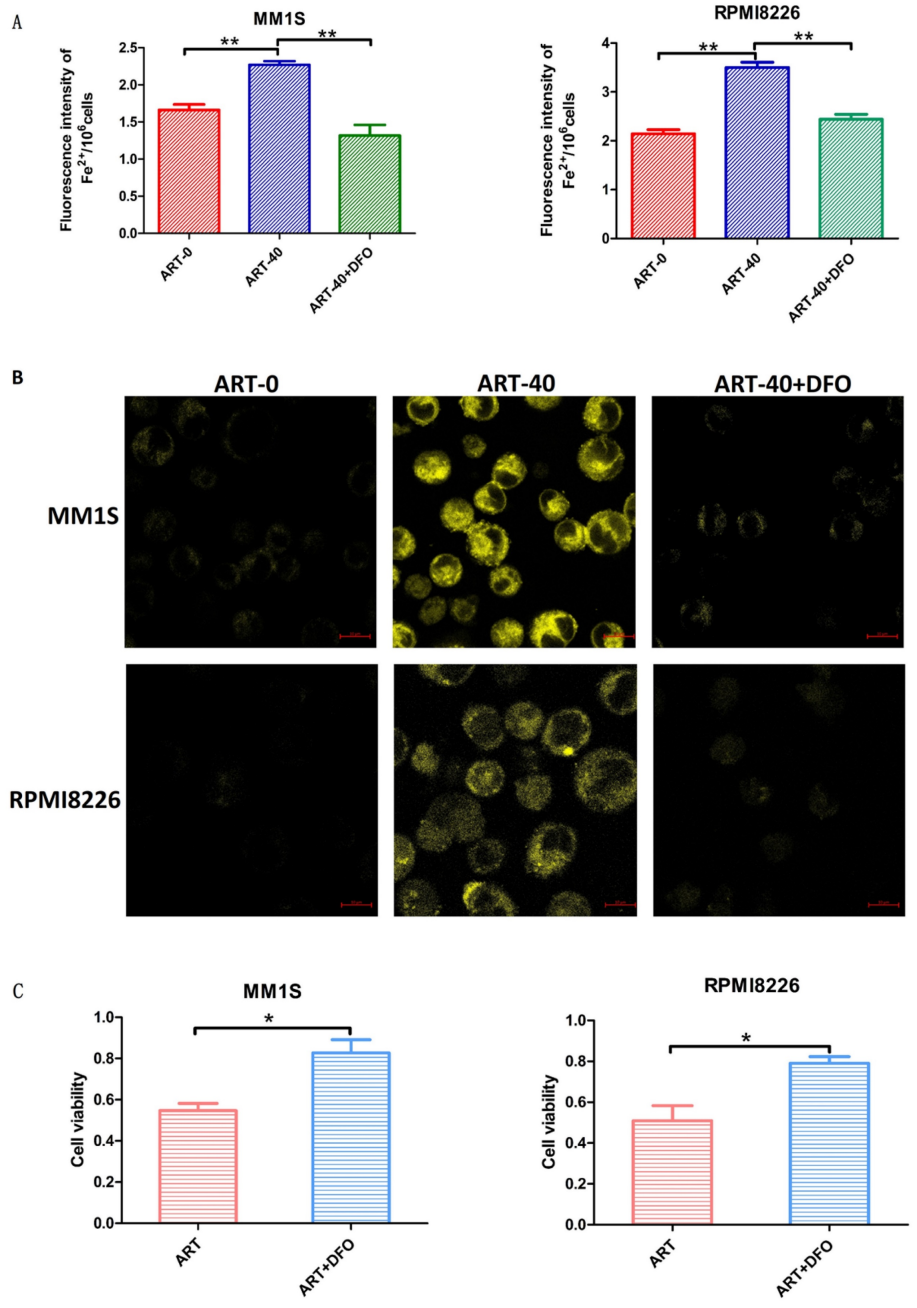
** Fe^2+^ levels in MM1S and RPMI8226 myeloma cells after different treatments.** MM1S and RPMI8226 were treated with 40 µM ART in the presence or absence of DFO (5 µM) for 48 h, then the levels of iron were measured using a fluorescent microplate reader (A) and a confocal microscope (B). Cell viability was determined using a CCK-8 assay (C). * P < 0.05, ** P < 0.01, compared with the ART group (40 μM), n=3. Data are presented as the mean ± SE.

Ferroptosis is a type of cell death accompanied by elevated levels of Fe^2+30^. To determine whether the ART-induced cell death in myeloma cells was caused by ferroptosis, we measured Fe^2+^ levels in the ART0, ART40 and ART40+DFO groups. According to Fig. 3 panels A and B, a cumulative increase in Fe^2+^ was observed in two myeloma cell lines after being treated with ART, and combination treatment with DFO could reverse this increase. Next, we measured cell viability to explore the relationship between the level of Fe^2+^ and ART-induced cell death. We found that DFO, which had a negative effect on Fe^2+^ levels, could rescue ART-induced cell death in myeloma cells (Fig. 3C). These results indicated that ART induces ferroptosis in myeloma cells. For cell lines resistant to ART, the concentration of Fe^2+^ did not show significant alteration after treatment in the same condition (Supplementary Fig. S3A, B).

### ART-induced changes in ROS and lipid peroxidation in myeloma cells

**Figure 4:**
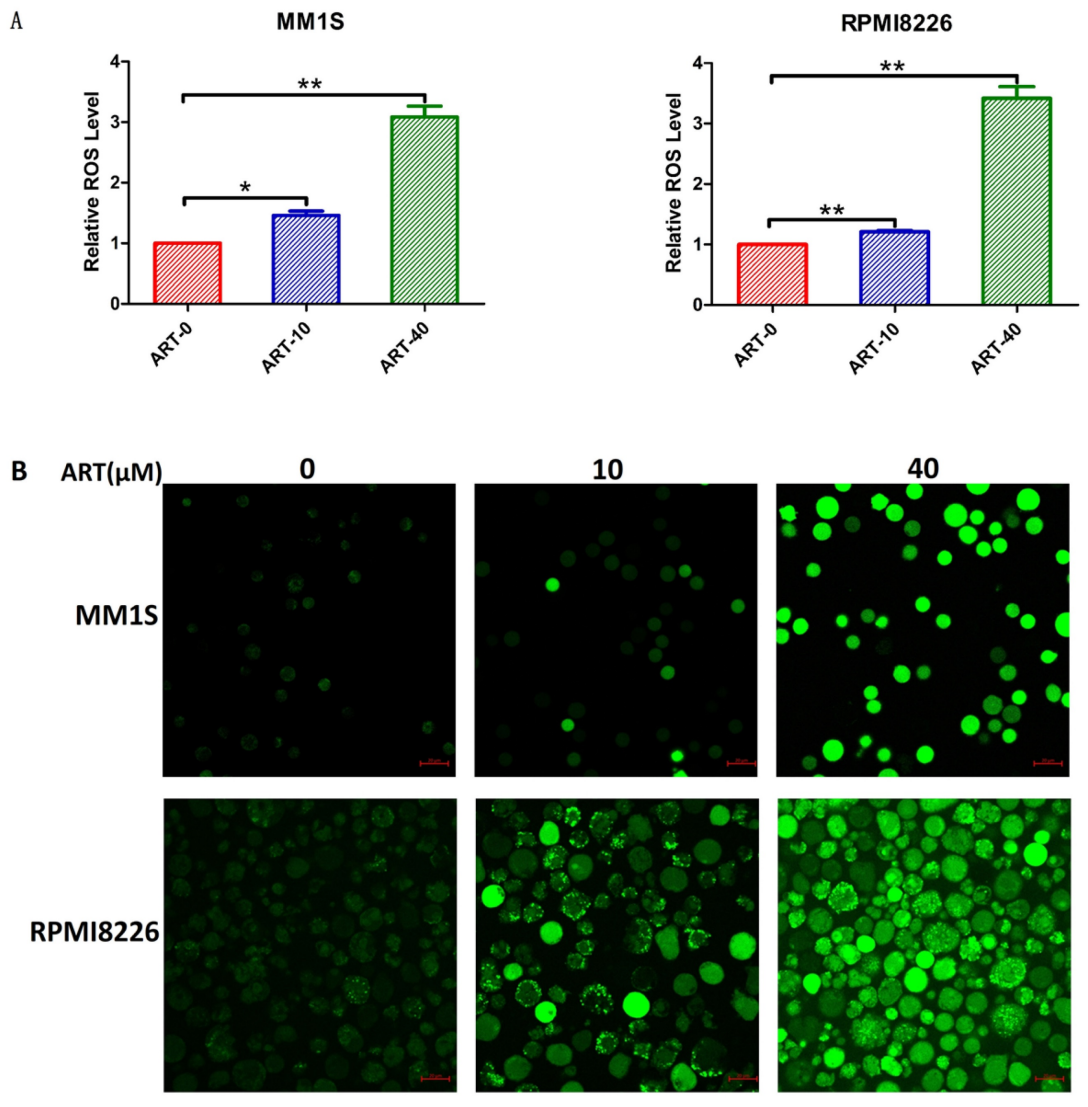
** Level of ROS in myeloma cells after treatment with ART.** MM1S and RPMI8226 cells were treated with different concentrations of ART for 48 h, and then the level of ROS was measured using a fluorescence microplate reader (A) and a confocal microscope (B). The relative levels of ROS measured by a fluorescence microplate reader were normalized to those measured in the ART0 group. * P < 0.05, ** P < 0.01, compared with the ART0 group, n=3.

**Figure 5:**
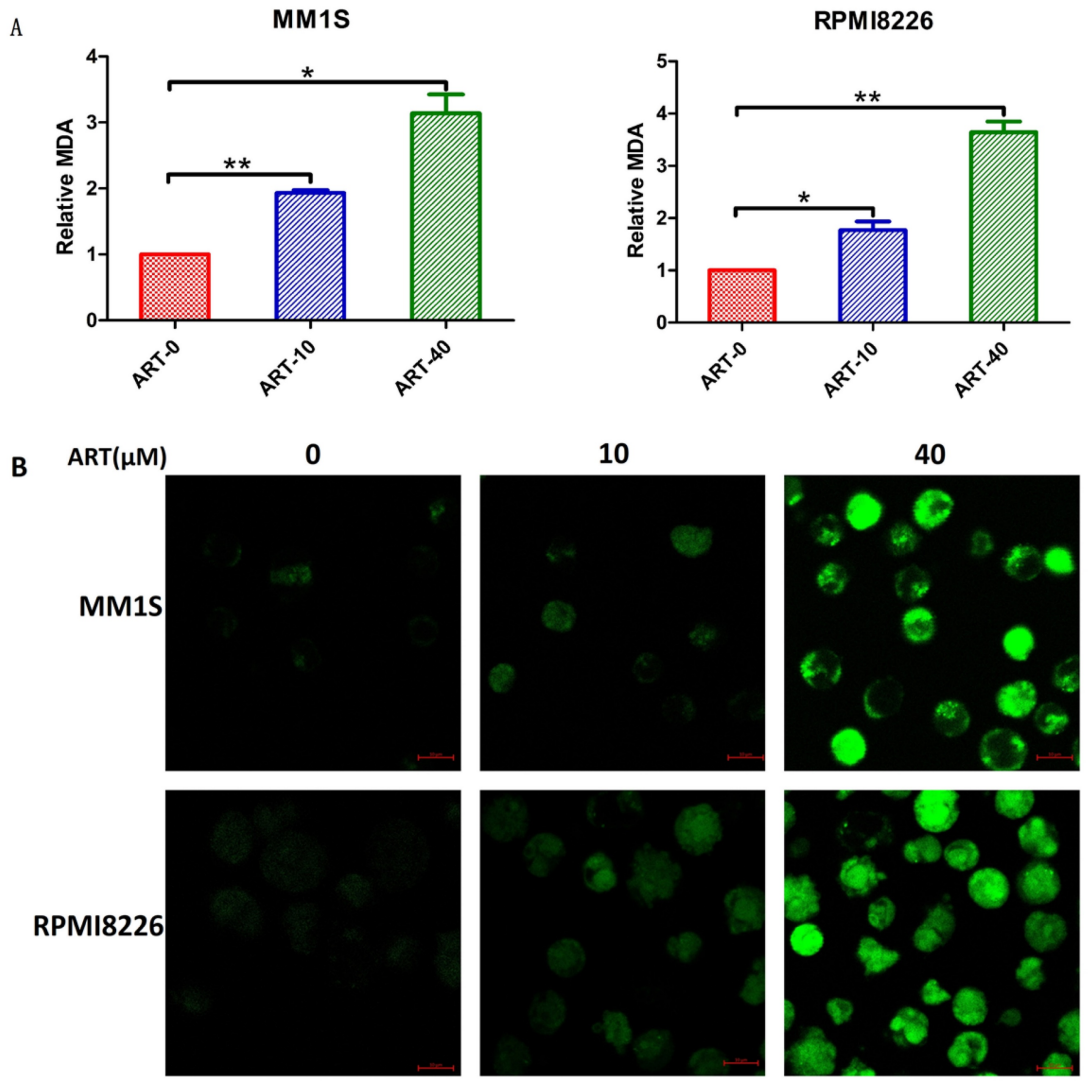
** Level of lipid peroxidation in myeloma cells after treatment with ART.** MM1S and RPMI8226 cells were treated with different concentrations of ART for 48 h, and then the MDA levels were measured as an indication of lipid peroxidation was measured according to MDA through a fluorescence microplate reader (A) and quantified by Liperfluo through confocal microscopy (B). The relative level of MDA measured by a fluorescence microplate reader was compared to that of the ART0 group. * P < 0.05, ** P < 0.01, compared with the ART0 group, n=3.

Elevated ROS levels and lipid peroxidation represent the destruction of the cell structure and membrane and are specific characteristics of ferroptosis 30. After confirming the effects of ART on cell viability and Fe^2+^ levels in myeloma cells, we explored the effects of ART on ROS production and lipid peroxidation. The results in Fig. 4 show that ART can induce a cumulative increase in ROS. Similar increases in the levels of MDA (Fig. 5A) and Liperfluo probe fluorescence (Fig. 5B), which represent the extent of lipid peroxidation, were also observed. For cell lines resistant to ART, the ROS or lipid peroxidation did not show significant alteration after treatment in the same condition (Supplementary Fig. S4,5 A,B).

### The effect of ART on the ferroptosis regulators *GPX4* and *ACSL4*

**Figure 6:**
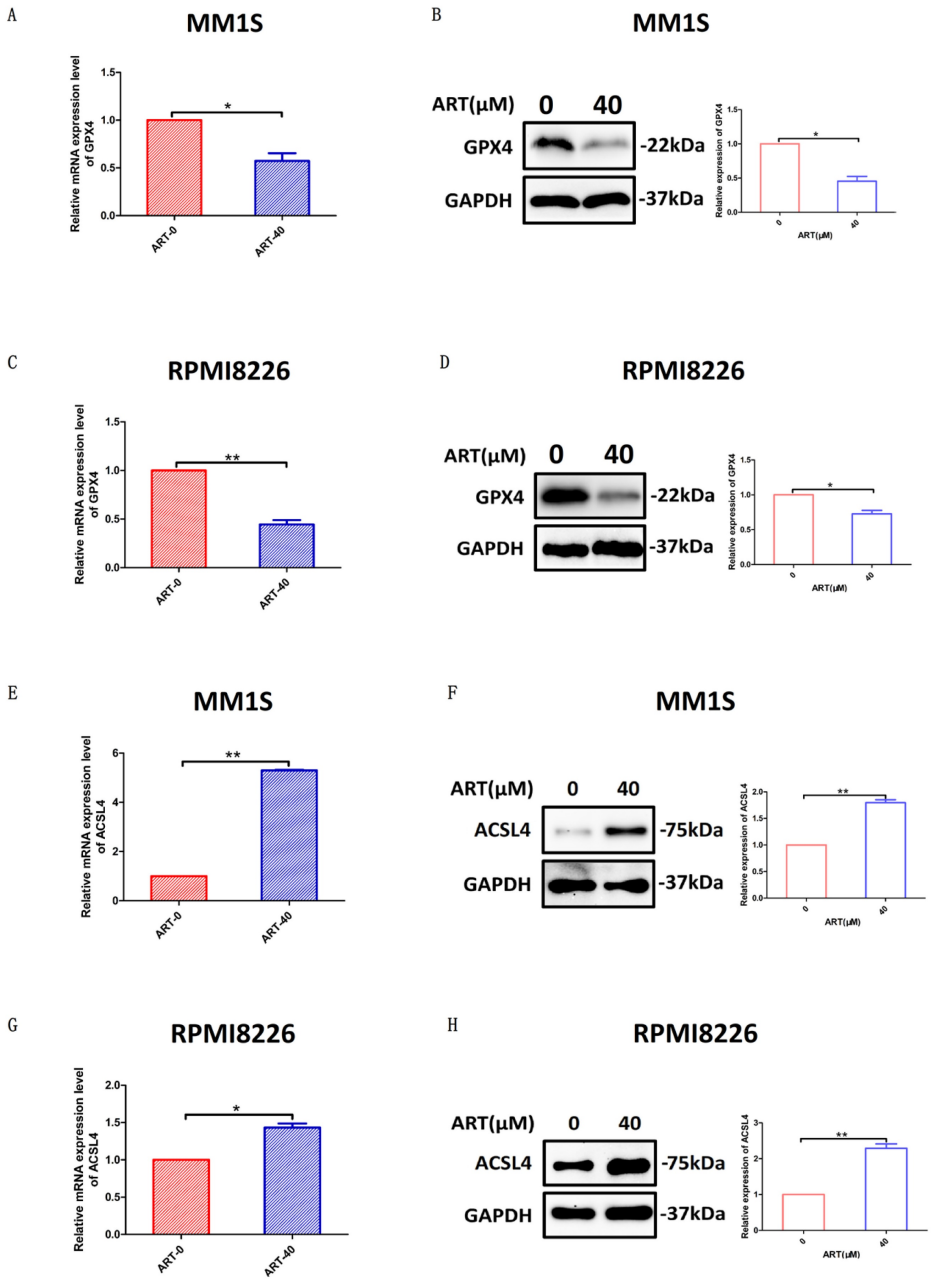
** The effect of ART treatment on the ferroptosis regulators *GPX4* and *ACSL4*.** MM1S and RPMI8226 cells were treated with ART (40 μM) for 48 hours (A-H). GPX4 (A, C) and ACSL4 (E, G) mRNA levels were assessed by qRT‒PCR (n = 3). GPX4 (B, D) and ACSL4 (F, H) protein levels were assessed by Western blotting (n = 3) * P < 0.05, ** P < 0.01. Data are presented as the mean ± SE.

Glutathione peroxidase 4 (GPX4) and ACSL4 are key regulators of ferroptosis^31^. GPX4 can directly inhibit ferroptosis^32^, while ACSL4 can positively regulate ferroptosis by promoting membrane lipid peroxidation^30^. To further confirm that ART had the capacity to induce a series of alterations related to ferroptosis, we sought to explore whether GPX4 and ACSL4 expression was affected in MM1S and RPMI8226 cells after treatment with ART. According to the results shown in Fig. 6, expression of GPX4 was reduced in the two myeloma cell lines, while ACSL4 expression increased. For cell lines resistant to ART, the expression of GPX4 or ACSL4 did not show significant alteration after treatment in the same condition (Supplementary Fig. S6A-H).

### ART-induced ferroptosis in MM cells via the SREBP2-IPP-GPX4 pathway

Our results have shown that ART can induce ferroptosis in MM1S and RPMI8226 cells at a specific concentration, but the mechanism by which this occurs is not clear. Previous studies have shown that ART can inhibit the nuclear localization of SREBP2 in glioma and can also inhibit the expression of metabolites belonging to the methylvalproic acid (MVA) pathway, such as HMGCR and isopentenyl pyrophosphate (IPP)^24^, ^33^, ^34^. IPP is the precursor of CoQ10 and is also a limiting substrate for enzymatic isopentenylation of Sec-tRNA, thereby affecting the expression of GPX4^33^. Therefore, we speculate that ferroptosis induced by ART in myeloma cells may occur via the regulation of SREBP2 nuclear localization and via the SREBP2-IPP-GPX4 pathway.

**Fig 7:**
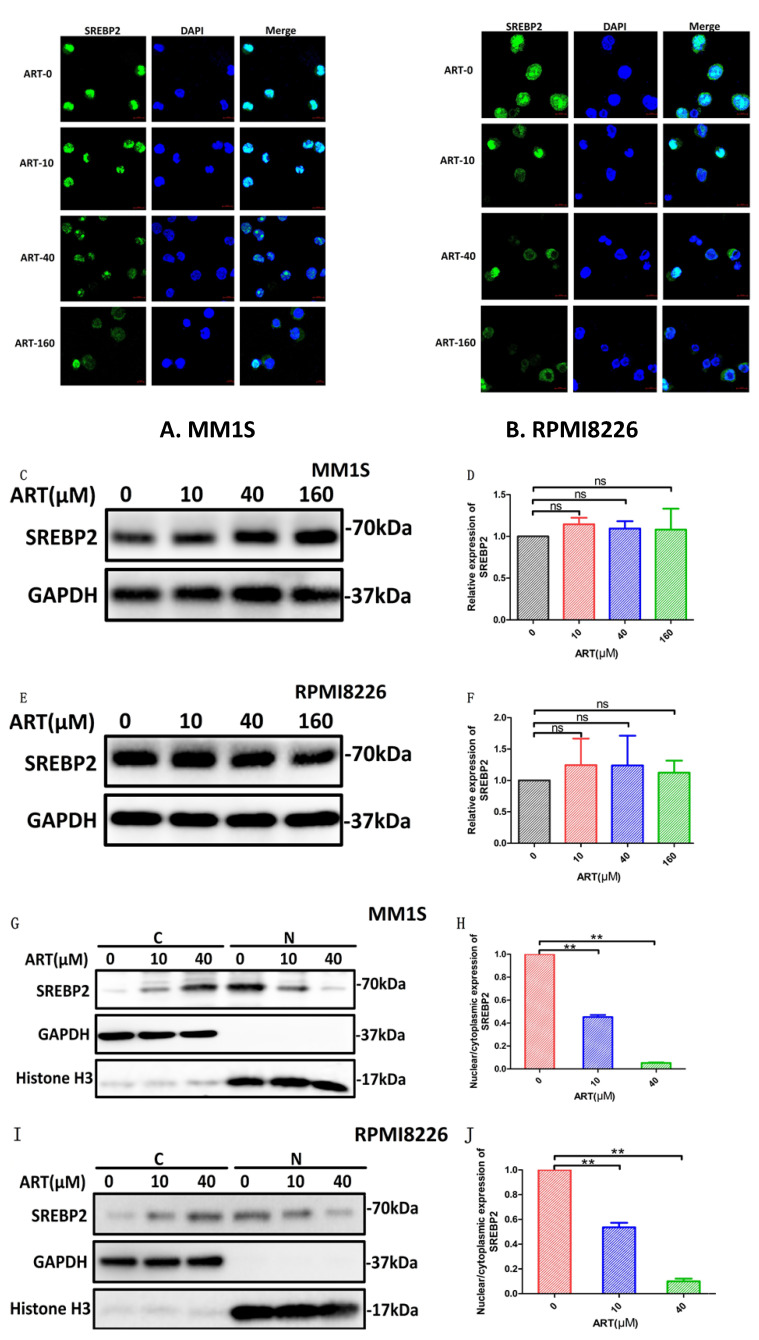
** The effect of ART treatment on SREBP2 nuclear localization in myeloma cells.** MM1S and RPMI8226 cells were treated with different concentrations of ART for 24 h, and alterations in the location of SREBP2 were observed by immunofluorescence staining (A,B) and by Western Blot(G,I). Alteration of the total SREBP2 protein levels were observed by Western Blot (C-F). Nuclear localization of SREBP2 in MM1S (H) and RPMI8226 (J) cells was calculated as a ratio of nuclear to cytoplasmic fluorescence using Image J. * P < 0.05, ** P < 0.01, compared with the ART0 group, n=3.

To explore the SREBP2-IPP-GPX4 pathway in ART-induced ferroptosis, we first observed changes in SREBP2 nuclear localization before and after ART treatment using a laser scanning confocal microscope. Compared with the blank group, the amount of SREBP2 localized to the nucleus was significantly reduced in MM1S and RPMI8226 cells after ART treatment (Fig. 7A, B). In order to confirm whether ART reduces only the nuclear localization of SREBP2 or, more generally, total SREBP2 protein levels, we subsequently detected the changes in the total expression level of SREBP2 treated by ART through Western blot. There was no significant change in the total amount of SREBP2 after ART treatment of myeloma cells (Fig. 7C-F). Then, we evaluated the effects of ART on the expression of SREBP2 in the cytoplasm and nucleus separately. As the concentration of ART increased, the content of SREBP2 in the cytoplasm increased, while the content in the nucleus decreased (Fig. 7G, I). This observation was also confirmed by a decreased nuclear:cytoplasmic ratio after ART administration compared to that in cells that were not administered ART (Fig. 7H,J), suggesting that ART mainly affects the nuclear localization of SREBP2 rather than the total amount of SREBP2. These results indicated that the nuclear localization of SREBP2 could be inhibited by ART.

**Figure 8:**
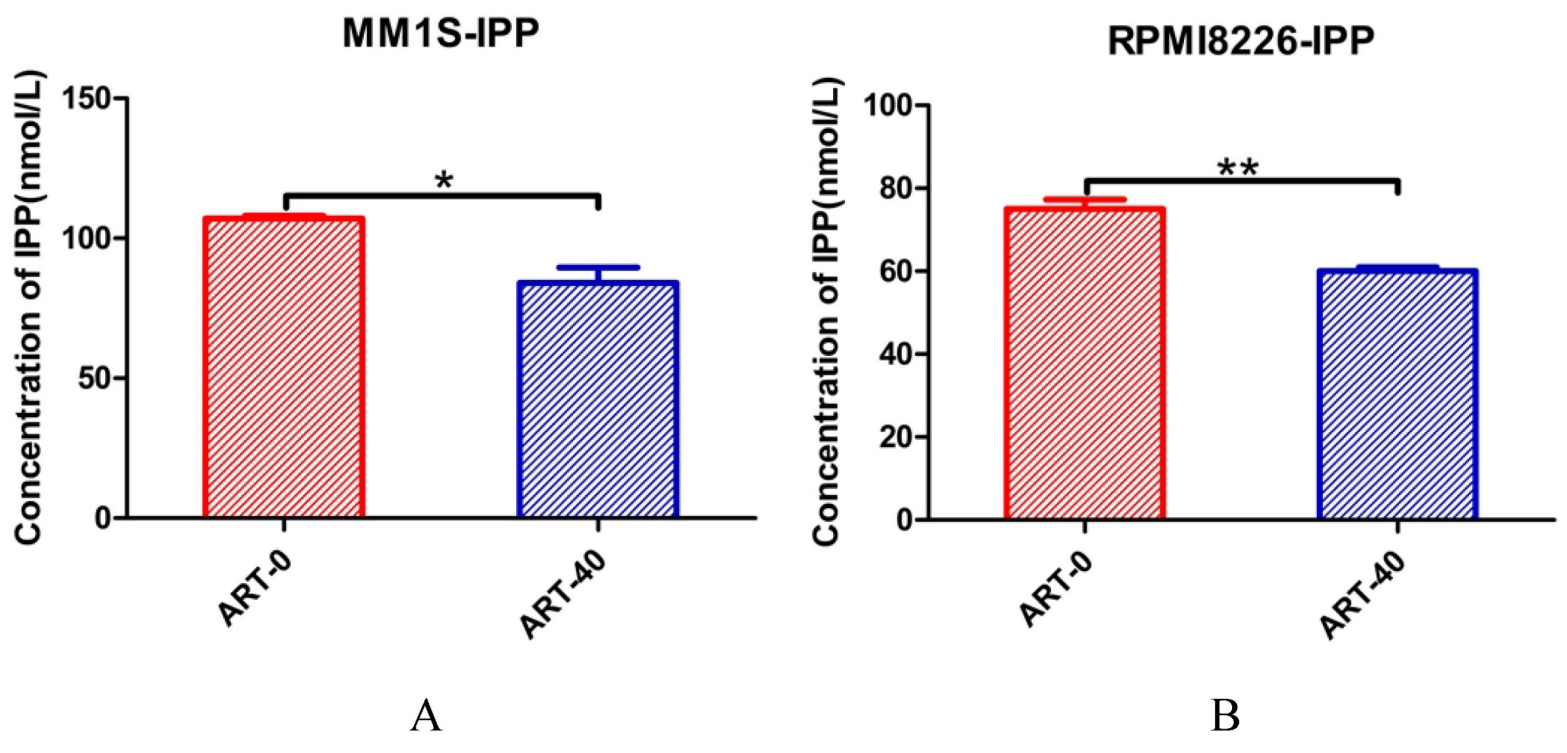
** The effect of ART treatment on the concentration of IPP in myeloma cells.** MM1S (A) and RPMI8226 (B) cells were treated with different concentrations of ART for 24 h, and then the level of IPP was measured using an ELISA kit and a microplate reader. * P < 0.05, ** P < 0.01, compared with the ART0 group, n=3.

**Figure 9:**
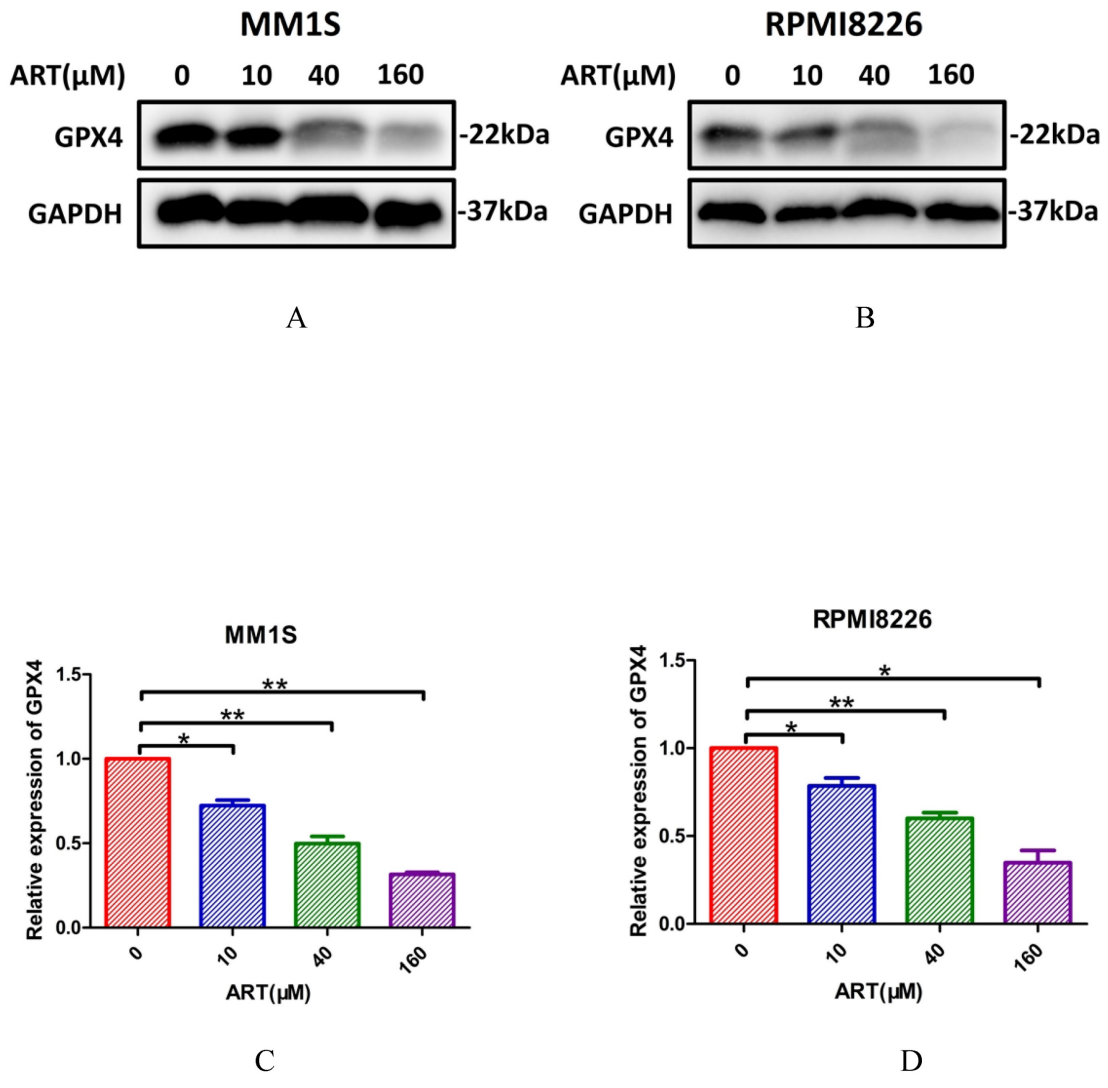
** The effect of ART treatment on the expression of GPX4 in myeloma cells.** MM1S (A and C) and RPMI8226 (B and D) cells were treated with different concentration of ART (0, 10, 40 and 160 µM) for 24 h, then the expression of GPX4 was assessed by Western blotting (n = 3)* P < 0.05, ** P < 0.01.

**Figure 10:**
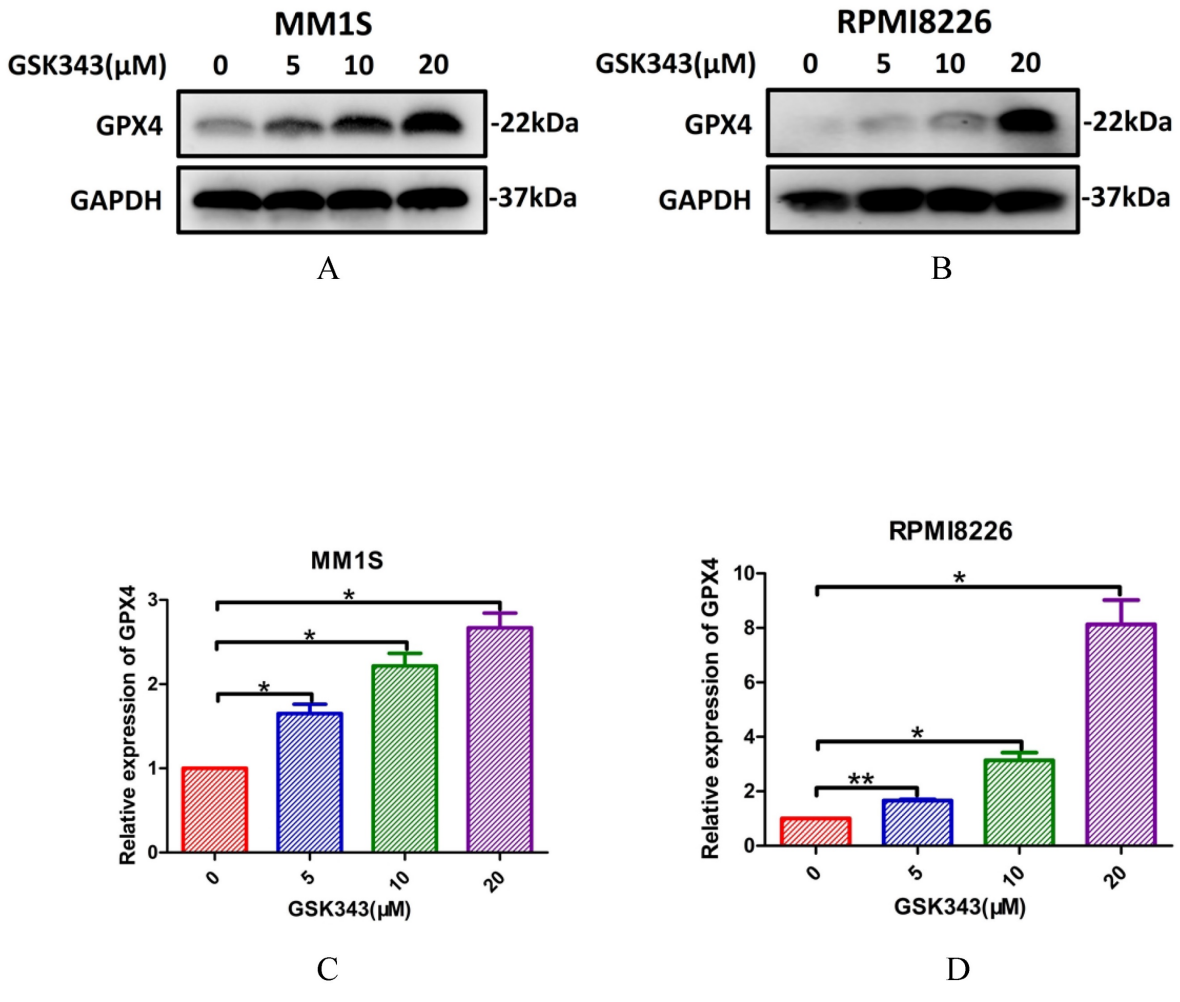
** The effect of GSK343 treatment on the expression of GPX4 in myeloma cells.** MM1S (A and C) and RPMI8226 (B and D) cells were treated with different concentrations of GSK343 (0, 5, 10 and 20 μM) for 6 h, following which the expression of GPX4 was assessed by Western blotting (n = 3)* P < 0.05, ** P < 0.01.

Next, we explored whether IPP levels may be affected by ART. The concentration of IPP in both myeloma cell lines decreased after ART treatment (Fig. 8). To assess if ART has a regulatory effect on GPX4 via the SREBP2-IPP-GPX4 pathway, we first evaluated the GPX4 expression in MM1S and RPMI8226 cells treated with ART at different concentrations. Expression of GPX4 gradually decreased with increasing ART concentration (Fig. 9). Then, we used GSK343^17^, a drug that has been reported to promote the nuclear localization of SREBP2, to determine whether ART treatment has a negative impact on the expression of GPX4. Fig. 10 shows that the expression level of GPX4 increased with increasing concentrations of GSK343, suggesting that interfering with SREBP2 nuclear localization can affect the expression of GPX4.

**Figure 11:**
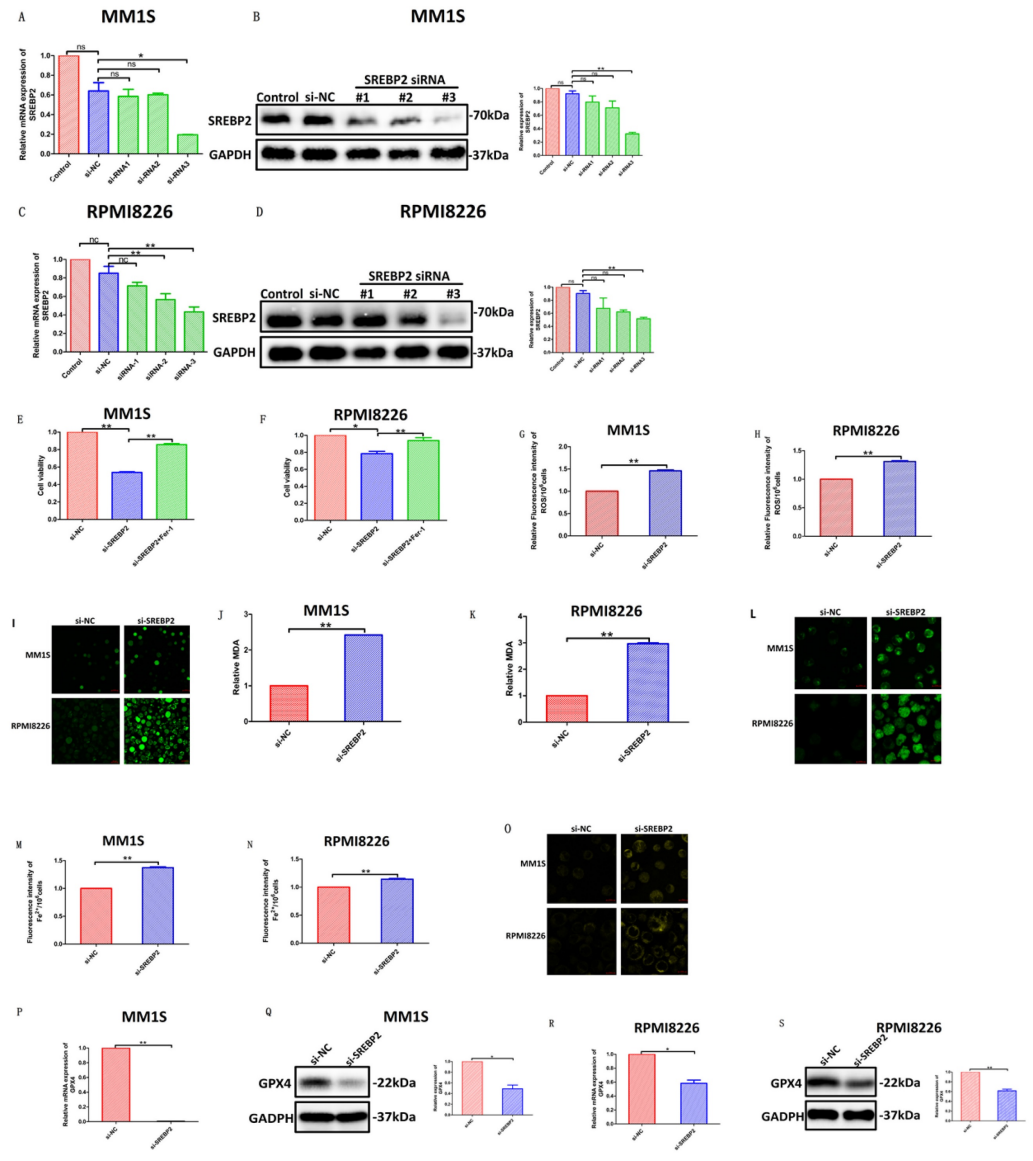
** Evaluation of the impact of transfection with si-SREBP2 in myeloma cells. (A-D)** qPCR (A,C) and Western blotting (B,D) of SREBP2 expression in myeloma cells transfected with SREBP2-siRNAs or negative control-siRNA. **(E,F)** Cell viability of myeloma cells transfected with negative control-siRNA, SREBP2-siRNA or SREBP2-siRNA +Fer-1, which was assessed by CCK-8 assay. **(G-I)** ROS of myeloma cells transfected with SREBP2-siRNA or negative control-siRNA, which was measured using a fluorescence microplate reader (G,H) and a confocal microscope (I). **(J-L)** Level of lipid peroxidation in myeloma cells transfected with SREBP2-siRNA or negative control-siRNA, which were according to MDA through a fluorescence microplate reader (J,K) and quantified by Liperfluo through confocal microscopy (L).** (M-O)** Fe^2+^ levels in myeloma cells transfected with SREBP2-siRNAs or negative control-siRNA, which were measured using a fluorescent microplate reader (M,N) and a confocal microscope (O).** (P-S)** qPCR (P,R) and Western blotting (Q,S) of GPX4 expression in myeloma cells transfected with SREBP2-siRNAs or negative control-siRNA.* P < 0.05, ** P < 0.01, compared with the si-NC group, n=3. Data are presented as the mean ± SE.

**Figure 12:**
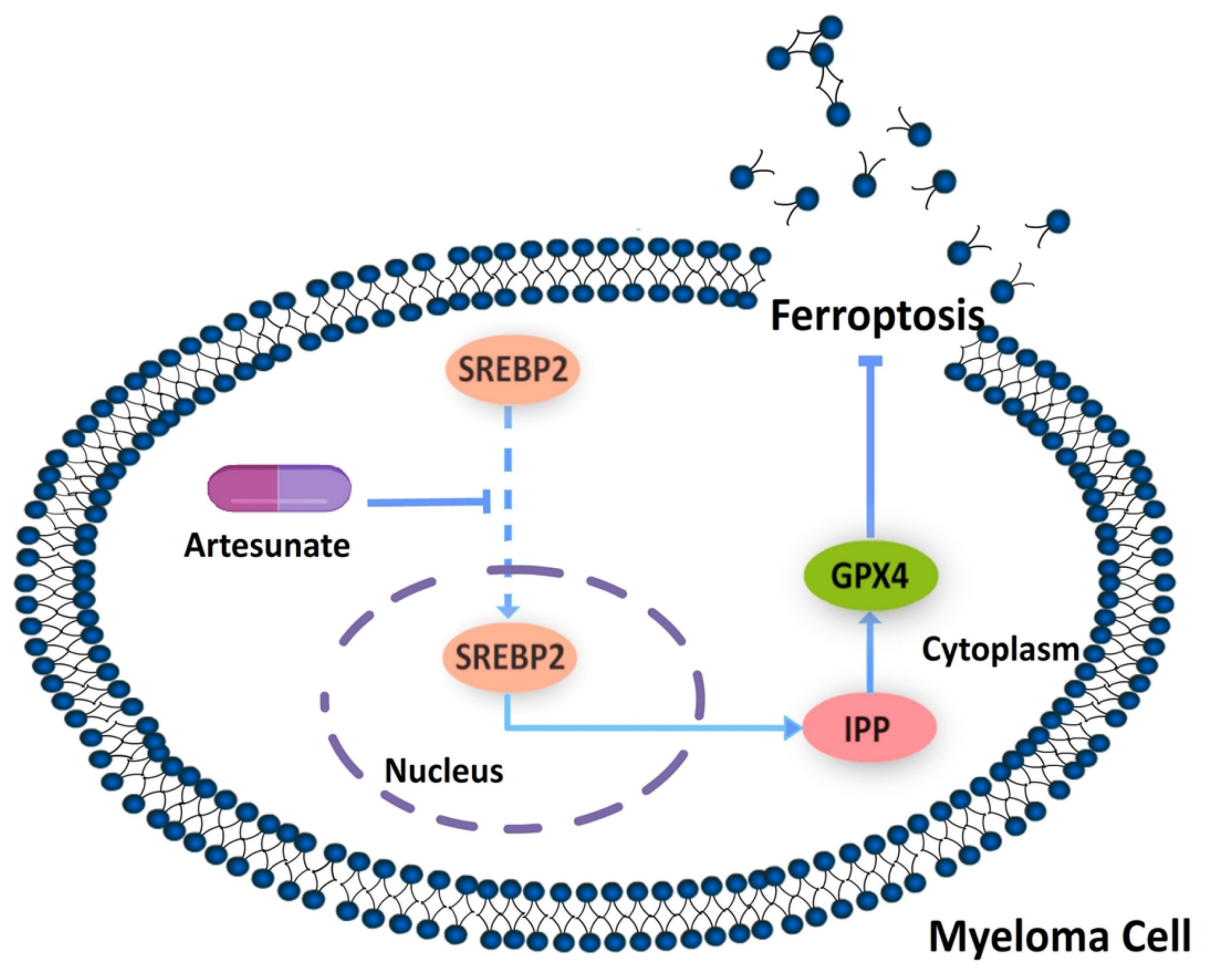
** Mechanism by which ART induces ferroptosis in myeloma cells.** Artesunate (ART) can induce inhibition of SREBP2 nuclear localization, followed by downregulation of IPP and GPX4, to eventually trigger ferroptosis in myeloma cells.

In order to further clarify the molecular mechanism of ART-mediated cell ferroptosis, we used siRNA to silence SREBP2 and detected the changes in various effects after silencing. We selected si-SREBP2-3, which has the most apparent knockout effect, as our primary means of silencing SREBP2 in the following experiments (Fig. 11A-D). We found that compared with cells transfected with control siRNA, silencing SREBP2 in myeloma cells resulted in a decrease in cell viability (Fig. 11E,F), which was, to some extent, rescued by Fer-1 (Fig. 11E,F). At the same time, the levels of ROS (Fig. 11G-I), Fe^2+^ (Fig. 11M-O) and the degree of lipid peroxidation (Fig. 11J-L) all increased in myeloma cells after being transfected with si-SREBP2. In addition, silencing SREBP2 can lead to downregulation of GPX4 (Fig. 11P-S), suggesting that under the treatment of si-SREBP2, the total amount of SREBP2 decreases, leading to a further decrease in its part located in the nucleus, thereby triggering a series of downstream effects. These results indicate that ART can induce ferroptosis in myeloma cells through the SREBP2-IPP-GPX4 pathway (Fig. 12).

## Discussion

MM is the second most common haematological malignancy after lymphoma^35^. MM is incurable and characteristically has poor prognosis^36^, making it necessary to explore new treatment approaches. In this study, we demonstrated that, in myeloma cells, ART treatment inhibited nuclear localization of SREBP2 and induced ferroptosis through the SREBP2-IPP-GPX4 axis, thereby inhibiting proliferation.

ART, a first-line drug against Plasmodium (the parasite that causes malaria), has shown promise in treating haematological malignancies in the past decade, especially for addressing drug resistance and treatment in leukaemia and lymphoma^25^. However, progress has been slow in MM^25^. Most previous studies on ART in myeloma cells have focused on role in promoting apoptosis^25^, ^27^, and few other types of cell death have been explored. In 2014, a study focused on ART and myeloma cells showed that some of the myeloma cells that had been treated with ART failed to reverse the inhibition of proliferation mediated by apoptosis inhibitors such as Z-VAD-mfk^29^, suggesting that ART had a potent ability to induce non-apoptotic cell death in myeloma cells. However, whether this type of non-apoptotic cell death was ferroptosis was not determined. Further studies have also noted that ART can induce an increase in intracellular Fe^2+^ levels in myeloma cells^29^, but there is a lack of evaluation of the inhibition of proliferation in myeloma cells after the application of iron chelators or the application of the ferroptosis-specific inhibitor Fer-1. These results prompted us to consider whether there were any other types of regulated cell death (RCD) associated with the use of ATR in myeloma cells, especially ferroptosis, which is Fe^2+^ dependent and is associated with changes in Fe^2+^ levels^30^. To assess this, we first treated myeloma cells with ART and then evaluated a series of ferroptosis-related features, including the rescue effect of various cell death inhibitors, alterations in ROS and lipid peroxidation levels, changes in Fe^2+^ levels and their effects on ART-induced cell death, and changes in markers of ferroptosis, such as* GPX4* and *ACSL4*. We found that at a specific concentration of ART, the inhibition of myeloma cell proliferation can be rescued by the ferroptosis-specific inhibitor Fer-1 but not by other types of cell death inhibitors. Furthermore, ROS, lipid peroxidation and Fe^2+^ levels were obviously increased after treatment with ART. In addition, ART-induced inhibition of myeloma cell proliferation can be rescued by DFO, which decreases the level of iron in cells. Moreover, both *GPX4* and *ACSL4* exhibited altered expression which was related to ferroptosis. All of these results indicate that ART has the potential to induce ferroptosis in myeloma cells.

Notably, when given the same treatment concentration of ART, apoptosis instead of ferroptosis happened in BEAS-2B and U2OS, which were resistant to ART. It suggests that ART may induce ferroptosis in specific cells. Holien et al. reported that ART can also induce apoptosis in myeloma 26. However, the myeloma cell lines used in that study were OH-2, IH-1, and INA-6, which differed from ours. Although they belong to the same myeloma cell line, their cellular genetic backgrounds may be different. Meanwhile, the concentration of ART that can produce apoptotic effects in that study was between 5-10 μM 26, which was lower than the concentration in our study (40 μM). However, human lymphoma cell lines DAUDI and CA-46 had been reported that triggered ferroptosis when treated with ART ranging from 5 to 10 μM in another study 37. These findings indicate that ART induces ferroptosis depending on specific cell lines and concentrations.

SREBP2 belongs to the same SREBP family subtype as SREBP1a and SREBP-1c. SREBP2 is composed of an amino-terminal, serine-rich, and proline-rich transcriptional activity domain, bHLH-LZ chain structure, two hydrophobic transmembrane segments and carboxyl terminus^38^. As one of the key regulators in lipid metabolism, SREBP2 can only perform its regulatory function when located in the nucleus^17^,^39^. In other words, alteration of its nuclear localization may affect its downstream regulation. With the known relationship between ART and the nuclear localization of SREBP2 in glioma, we speculated that the nuclear localization of SREBP2 regulated by ART in myeloma cells may impact the ferroptosis. Through a series of experiments, we found that the nuclear localization of SREBP2 was inhibited and the expression levels of GPX4 and IPP were decreased when cells treated with ART were compared to the control group. The converse alteration of GPX4 expression was observed when cells were treated with GSK343, to promote the nuclear localization of SREBP2^17^. Together, these results indicate that ART can induce ferroptosis through the SREBP2-IPP-GPX4 pathway in myeloma cells.

However, this study still has some limitations. First, although the distinction of ART-induced ferroptosis and apoptosis was observed at one particular ART concentration, other rare nonapoptotic types of RCD, such as copper-induced death, were not examined^39^. Previous studies have noted that changes in GPX4 levels may be closely related to copper death^39^. Second, when considering ferroptosis-related gene-level changes, we only investigated the expression of GPX4 and ACSL4 and did not examine other related genes, such as FTH1^40^ and PTGS2^30^. We will explore these in follow-up research.

## Conclusion

Collectively, our study demonstrates that ART can induce ferroptosis in myeloma cells by inhibiting nuclear localization of SREBP2. Through this study, we hope to establish a correlation between nuclear localization pathways and mediation of ferroptosis in myeloma cells and provide an innovative direction for the exploration of related therapies.

## Supplementary Material

Supplementary figures.
