# Supplementary material for: Artesunate induces ferroptosis by inhibiting the nuclear localization of SREBP2 in myeloma cells
Source: Int J Med Sci. 2023 Sep 18;20(12):1535–50. doi: 10.7150/ijms.86409 (PMC10583180; doi:10.7150/ijms.86409)
Supplement: Supplementary file 1 — Supplementary figures. [file ijmsv20p1535s1.pdf]

Supplemental data

Artesunate induces ferroptosis by inhibiting the nuclear localization of SREBP2 in myeloma cells

By Liang et al (MS ID: 86409y1)

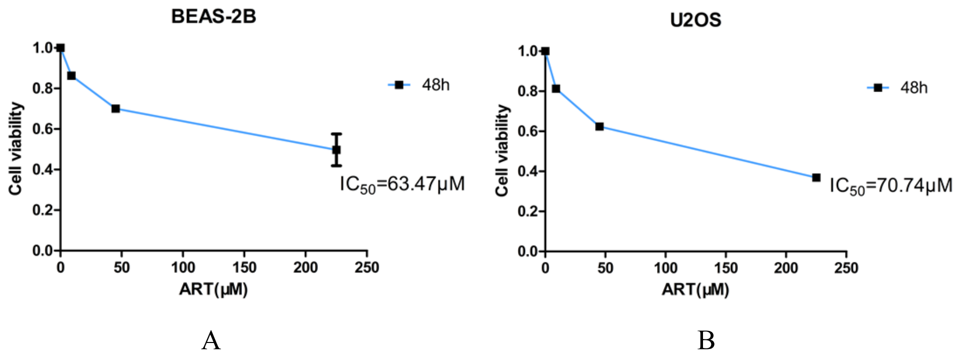

Supplemental Figure 1. IC_{50} for 48h ART exposure for the BEAS-2B (A) and U2OS (B) cell lines. Cells were treated with ART for 48 h, and cell viability was assessed using the CCK-8 assay.

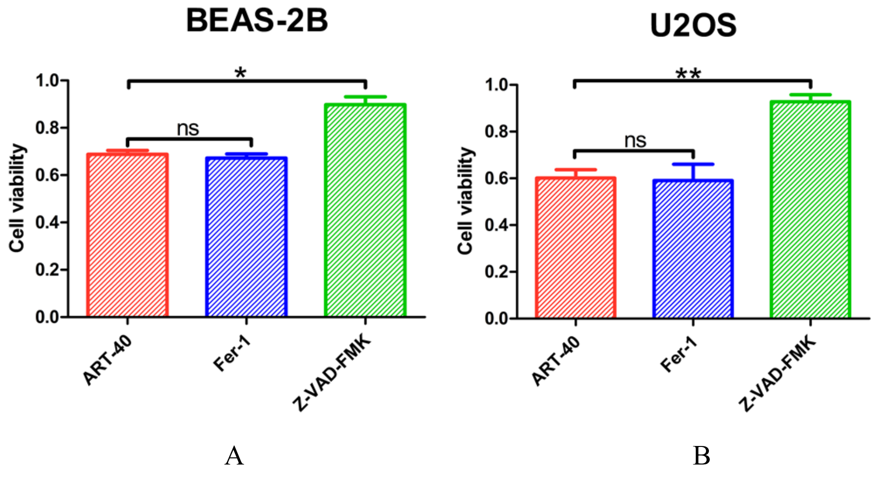

Supplemental Figure 2. Evaluation of the impact of treatment with different specific cell death inhibitors on ART-induced cell death in cells. BEAS-2B (A) and U2OS (B) were treated with ART (40 μM) in combination with Z-VAD-FMK (10 μM), Fer-1 (2.5 μM) for 48 h, then cell viability was assessed by CCK-8 assay. * $P < 0.05$, ** $P < 0.01$, compared with the ART group (40 μM), n=3. Data are presented as the mean ± SE.

A

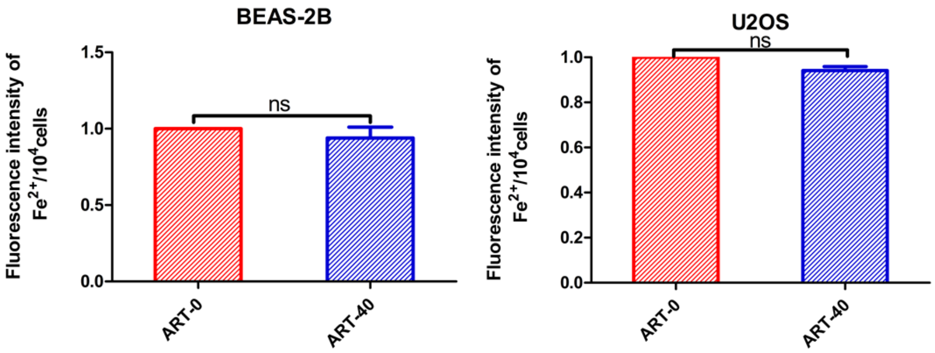

B

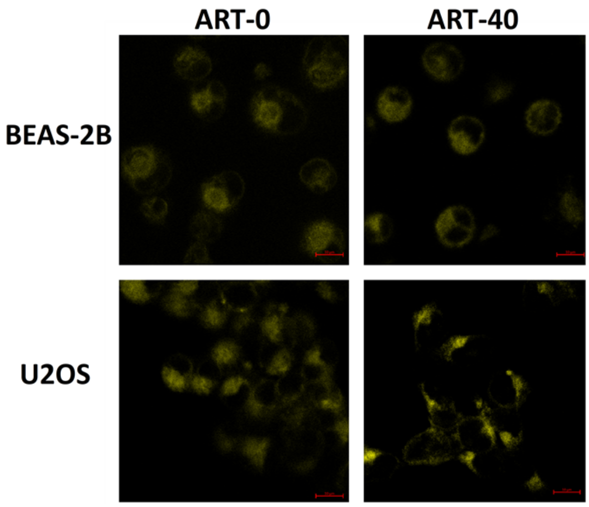

Supplemental Figure 3. Fe^{2+} levels in BEAS-2B and U2OS after different treatments. BEAS-2B and U2OS were treated with 40 μM ART or DMSO for 48 h, then the levels of iron were measured using a fluorescent microplate reader (A) and a confocal microscope (B).* $P < 0.05$, ** $P < 0.01$, compared with the DMSO group (40 μM), n=3. Data are presented as the mean ± SE.

A

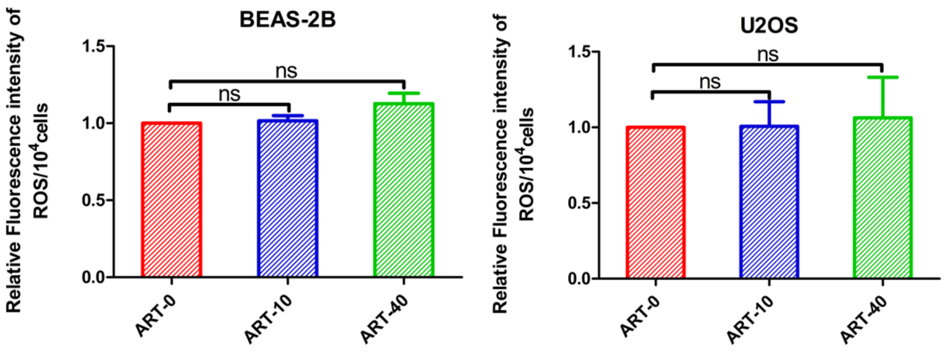

B

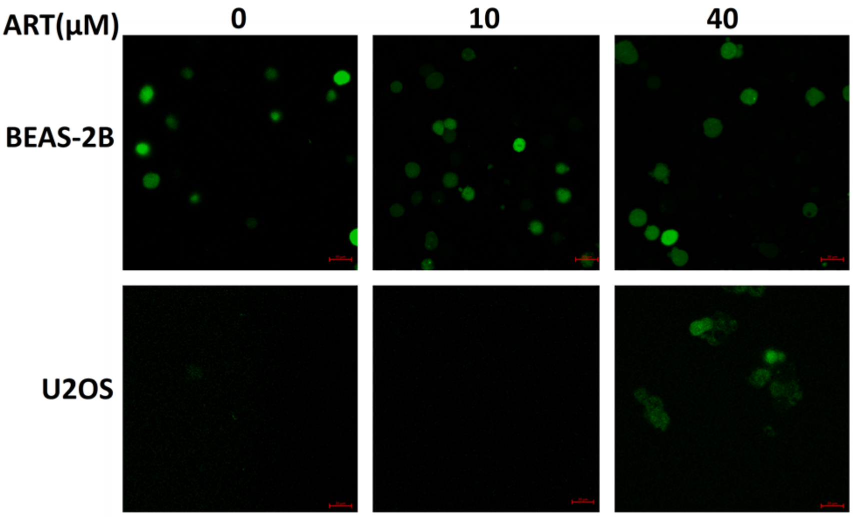

Supplemental Figure 4. Level of ROS in cells after treatment with ART. BEAS-2B and U2OS were treated with different concentrations of ART for 48 h, and then the level of ROS was measured using a fluorescence microplate reader (A) and a confocal microscope (B). The relative levels of ROS measured by a fluorescence microplate reader were normalized to those measured in the ART0 group. * $P < 0.05$, ** $P < 0.01$, compared with the ART0 group, n=3.

A

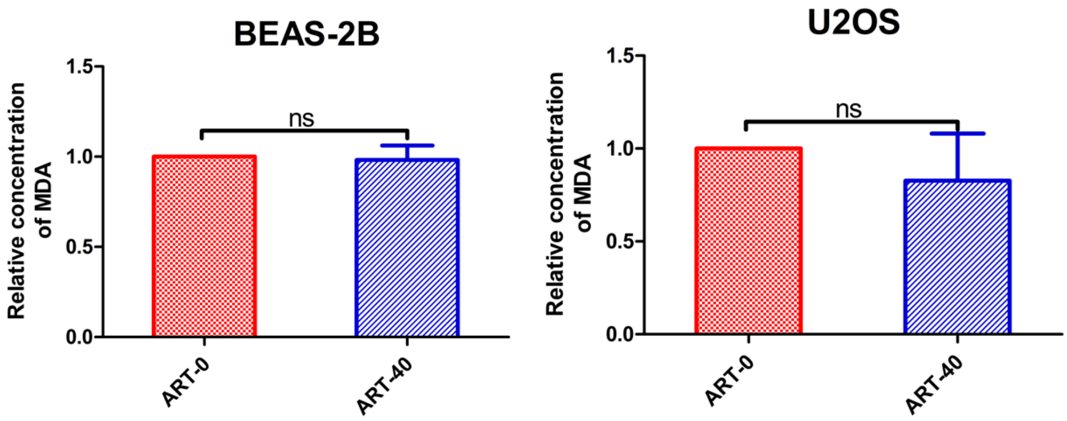

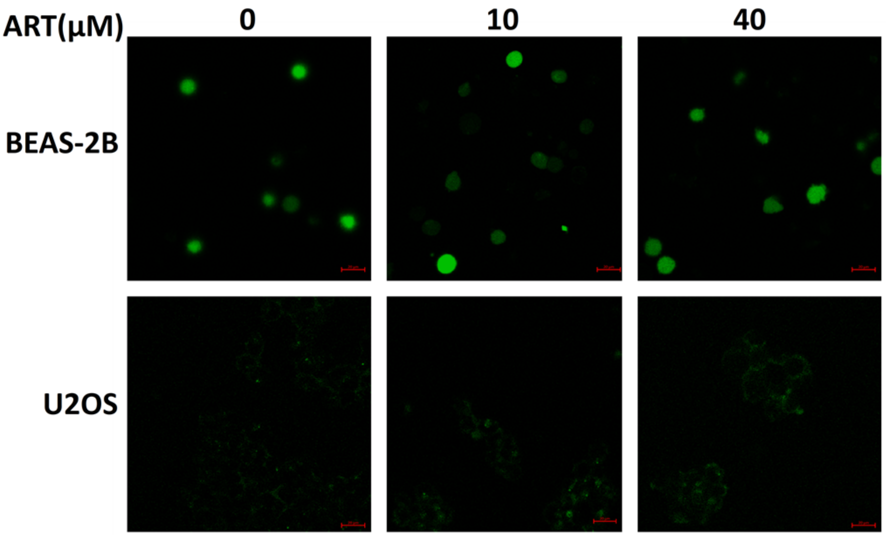

B

Supplemental Figure 5. Level of lipid peroxidation in cells after treatment with ART. BEAS-2B and U2OS were treated with different concentrations of ART for 48 h, and then the MDA levels were measured as an indication of lipid peroxidation was measured according to MDA through a fluorescence microplate reader (A) and quantified by Liperfluo through confocal microscopy (B). The relative level of MDA measured by a fluorescence microplate reader was compared to that of the ART0 group. * $P < 0.05$, ** $P < 0.01$, compared with the ART0 group, n=3..

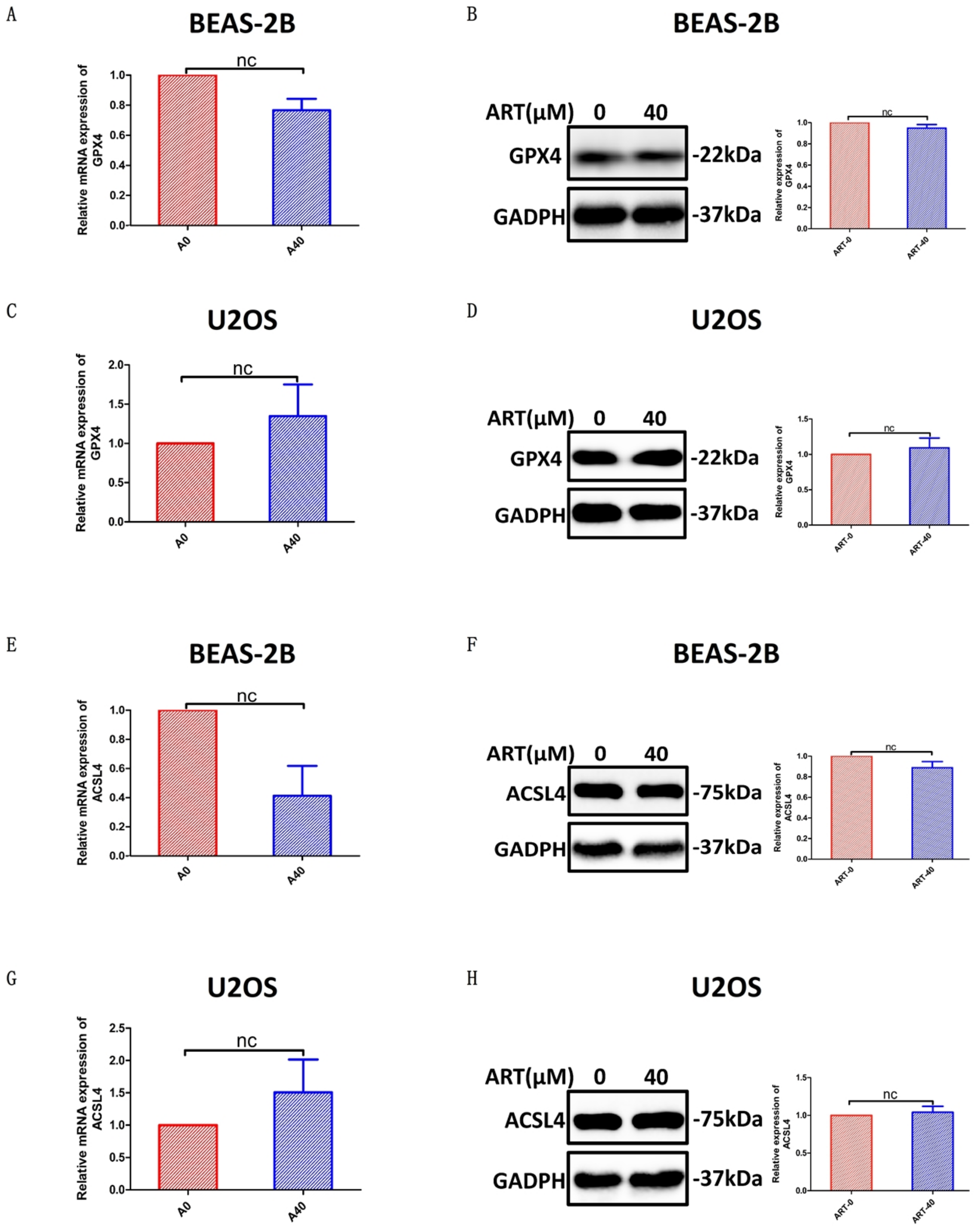

Supplemental Figure 6. The effect of ART treatment on the ferroptosis regulators *GPX4* and *ACSL4*.

BEAS-2B and U2OS were treated with ART (40 μM) for 48 hours (A-H). GPX4 (A,C) and ACSL4 (E, G) mRNA levels were assessed by qRT–PCR (n = 3). GPX4 (B, D) and ACSL4 (F, H) protein levels were assessed by Western blotting (n = 3) * P < 0.05, ** P < 0.01. Data are presented as the mean ± SE.
